# Real world evidence on gemcitabine and nab-paclitaxel combination chemotherapy in advanced pancreatic cancer

**DOI:** 10.1186/s12885-018-5244-2

**Published:** 2019-01-08

**Authors:** Hakon Blomstrand, Ursula Scheibling, Charlotte Bratthäll, Henrik Green, Nils O. Elander

**Affiliations:** 1 Department of Oncology, Ryhov County Hospital, 55305 Jönköping, Sweden; 2 Division of Oncology, Department of clinical and experimental medicine, Faculty of medicine and health sciences, Linköping University, 58183 Linköping, Sweden; 3 Department of Oncology, Kalmar County Hospital, 39244 Kalmar, Sweden; 4 Division of Drug Research, Department of Medical Health Sciences, Linköping University, 58183 Linköping, Sweden; 5 Department of Forensic Genetics and Forensic Toxicology, National Board of Forensic Medicine, 58758 Linköping, Sweden

**Keywords:** Pancreatic cancer, Chemotherapy, Gemcitabine, Nab-paclitaxel, Bone marrow toxicity

## Abstract

**Background:**

In the recent phase III trial MPACT the combination of gemcitabine and nab-paclitaxel (Gem/NabP) showed increased overall survival compared to gemcitabine alone in the treatment of advanced pancreatic ductal adenocarcinoma (aPDA). Until now there has been limited information on the clinical benefit and toxicity of the combination regimen in a real world setting. In addition the value for patients with locally advanced rather than metastatic aPDA has been unclear, since the former category of patients was not included in the MPACT trial.

**Methods:**

A multicentre retrospective observational study in the South Eastern Region of Sweden was performed, with the first 75 consecutive patients diagnosed with aPDA (both locally advanced and metastatic disease) who received first-line treatment with Gem/NabP.

**Results:**

In the overall population median progression free survival (PFS) and overall survival (OS) were 5.2 (3.4–7.0 95% CI) and 10.9 (7.8–14.0 95% CI) months, respectively. Patients with metastatic disease displayed a median OS of 9.4 (4.9–13.9) and a median PFS of 4.5 (3.3–5.7) months whereas the same parameters in the locally advanced subgroup were 17.1 (7.6–26.6) and 6.8 (5.2–8.4) months, respectively. Grade 3–4 hematologic toxicity was recorded: Neutropenia, leukopenia, thrombocytopenia, and anaemia were observed in 23, 20, 5, and 4% of patients, respectively. Dose reductions were performed in 80% of the patients.

**Conclusion:**

This study confirms the effectiveness and safety of first-line Gem/NabP in both locally advanced and metastatic PDA in a real world setting.

**Electronic supplementary material:**

The online version of this article (10.1186/s12885-018-5244-2) contains supplementary material, which is available to authorized users.

## Background

Pancreatic cancer is a severe disease with increasing incidence and high mortality, being the fourth most frequent cause of cancer related death in Sweden [1], Europe, and the US [2]. Symptoms are initially scarce which means that diagnosis is often delayed, and the majority of patients already have locally advanced or metastasised disease at the time of diagnosis [1, 3]. At this stage no surgery or other treatment with intention to cure is possible and prognosis without treatment is poor with median overall survival (OS) of less than six months and 5-year survival of less than 5% [1, 4].

Since the ground breaking trial by Burris et al. in 1997 [5], where gemcitabine was proven superior to 5-fluorouracil based therapy, gemcitabine monotherapy has remained the gold standard in the treatment of advanced pancreatic ductal adenocarcinoma (aPDA). Subsequent trials over the last 20 years have assessed the additional value of gemcitabine in combination with other traditional chemotherapeutics, targeted drugs, or both. However, the benefit of such combination regimens has been non-existent or very limited, and usually at the expense of increased toxicity [6–12].

The situation was altered by the recent phase III MPACT trial that showed nearly two months of increased median OS, from 6.7 to 8.5 months, with the nano albumin bound (nab)-paclitaxel/gemcitabine (Gem/NabP) combination regimen compared with gemcitabine alone [13]. On the other hand the Gem/NabP regimen was associated with significantly increased toxicity; e.g. the incidence of leukopenia was doubled and the incidence of febrile neutropenia was tripled with the combination regimen compared to gemcitabine alone [13]. In Sweden Gem/NabP was approved for aPDA by Dec 2014 and has since been the standard treatment for patients with ‘good’ performance status (equalling ECOG performance status 0–1). To what extent the efficacy and toxicity data from the phase III trial corresponds to what is achieved in ‘real-world’ patients remains to be elucidated since no phase IV studies have been conducted, and real world studies with sufficient follow up times are still limited. Neither has the outcome of Gem/NabP in patients with locally advanced rather than metastasised disease been addressed. This study was therefore designed to evaluate the effects and toxicity of Gem/NabP treatment of aPDA in a real world setting.

## Methods

### Patients

A retrospective observational study was conducted in the South East Region of Sweden, covering a population of approximately 1,050,000 citizens, involving the oncology departments of Jönköping, Kalmar, and Linköping. The study was approved by the Regional Ethics Review Board in Linköping (diary number 2017/110–31). At the participating sites all prescription of chemotherapy is covered by the digital software CSAM Cytodose (CSAM Health AS, Oslo, Norway). Therefore all eligible patients were identified with this software. Inclusion criteria were aPDA, including locally advanced as well as metastatic disease, and the administration of first line palliative treatment with Gem/NabP, with treatment starting between the approval of the regimen in December 2014 and March 2017. Histology based diagnosis was preferred but, when absent, diagnosis based on combinations of cytology, imaging, and/or serum tumour marker (CA19–9) was accepted. Treatment pause and follow up with slight progression allowing for restart of the same therapy was not considered as second line therapy. Neither was toxicity related de-escalation to single drug regimen (i.e. Gem/NabP to gemcitabine) considered second line therapy, unless clinical and/or radiological signs of progressive disease were evident. Concomitant treatment with other modalities and treatment beyond progression with other chemotherapy regimens were allowed. Patients that received Gem/NabP in other settings than first line palliative treatment or with other histology than PDA were excluded.

At the three centres Gem/NabP was administered in a similar way by intravenous infusion at days 1, 8, and 15 in a four week cycle at doses of 1000 mg/m^2^ and 125 mg/m^2^ for gemcitabine and nab-paclitaxel, respectively. Bone marrow toxicity was monitored with blood samples prior to each dose and treatment response was usually evaluated with computed tomography every 2–3 cycles.

Patient and tumour characteristics, baseline biochemistry, performance status, and CA19–9 as well as treatment data were extracted from medical records using a standardised case report form. All patients’ medical records were followed until date of death, or no later than 27th of Sep 2017, when follow-up period was closed. Primary endpoint was OS, which was defined as time from start of treatment until date of death or last follow-up. Secondary endpoints were median progression free survival (PFS), occurrence and grade of bone marrow toxicity, number of treatment cycles, dose intensity, and presence of second line therapy. PFS was defined as time from start of treatment until progression, either radiological or clinical, or death, whichever came first.

The present study was conducted and reported in accordance with the STROBE guidelines (http://www.strobe-statement.org/).

### Statistical analysis

All analyses were carried out in the intention to treat population (which in this case was all patients that were prescribed at least one dose of Gem/NabP). Patient characteristics and tumour and treatment data are reported as frequencies and percentage for categorical variables and median with 95% confidence interval and range for continuous variables. Median OS and PFS for the whole cohort and subgroups (locally advanced versus metastatic disease, metastatic burden: more or less than 3 metastases, and performance status 0 versus 1–2) were estimated using Kaplan-Meier survival analyses and the significance of the difference in estimates of median survival with 95% confidence interval was calculated using log rank test. Cox regression analysis was used to evaluate hazard ratios for the same subgroups. Analyses were performed using SPSS v24 (IBM Corp. Armonk NY).

## Results

**Fig. 1:**
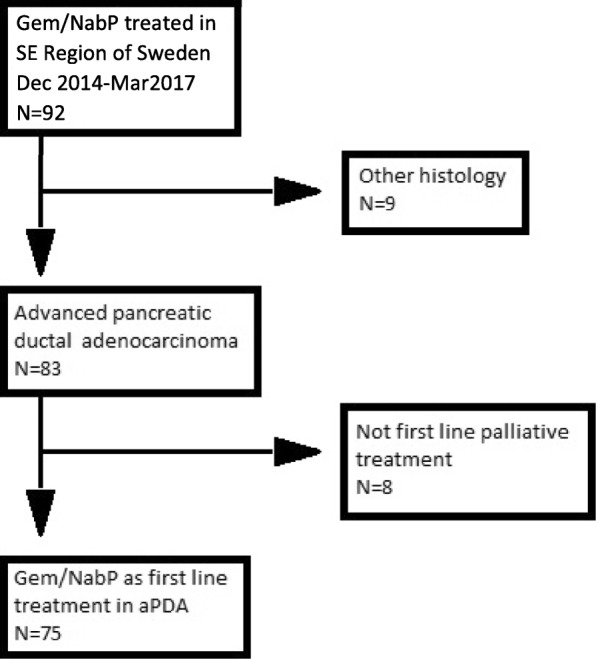
Flowchart for inclusion and exclusion criteria. Final cohort consisted of 75 patients. Nine patients were excluded due to ‘other histology’, which in this case was primary cancer of the ovaries (*n* = 3), biliary tract (*n* = 2), papilla Vateri (n = 2), leiomyosarcoma (*n* = 1), and oesophagus (n = 1). Eight patients were excluded due to Gem/NabP not given as first line treatment; these had either received FOLFIRINOX (*n* = 4) or gembitabine (n = 1) first line, never started Gem/NabP (n = 2), or received Gem/NabP as third line treatment (n = 1)

A total of 92 unique patients treated with Gem/NabP were identified. Of these patients 17 were excluded based on the exclusion criteria, leaving a total cohort of 75 patients representing the final study cohort (Fig. 1).

Patient and tumour characteristics are displayed in Table 1. PDA diagnosis was based on histology in 63 patients (84%). 71% of the patients had metastasised disease and 70% of those had more than three metastases. Liver (50%), lung (28%), and peritoneum (8%) were the most frequent metastatic sites. The distribution of ECOG performance status was 44, 48, and 8% for performance status 0, 1 and 2, respectively (Table 1). 35% of the patients had previously received chemotherapy in a non-palliative setting (24 patients with adjuvant and 3 patients with neoadjuvant treatment, respectively). None of the patients in this study received radiotherapy. Published data from the MPACT [13] study are listed to the far right in Table 1 for comparison. Due to the different inclusion criteria and patient characteristics no quantitative comparisons between the present population and the MPACT trial population were performed.

**Table 1:** Patient and treatment characteristics

	SE Region	MPACT (Gem/NabP-arm)
Total number	75 (100)	431 (100)
Gender		
Female	34 (45)	186 (43)
Male	41 (55)	245 (57)
Age median(range)	66 (48–80)	62 (27–86)
Distribution		
< 65	29 (39)	254 (59)
≥ 65	46 (61)	177 (41)
Length cm median(range)	172 (150–192)	
Weight kg median(range)	72 (41–121)	
Body surface kg/m^2^ median(range)	1.88 (1.4–2.37)	
ECOG Performance status		
0	33 (44)	69 (16)
1	36 (48)	328 (76)
2	6 (8)	32 (7)
CA19–9 U/ml median(range)	593.5 (1–140,000)	2293 (1.9–6,159,230)
Tumour stage		
Locally advanced	22 (29)	0 (0)
Metastasised	53 (71)	431 (100)
Number of metastases		
1	8 (15)	33 (8)
2	6 (11)	202 (47)
3	2 (4)	136 (32)
> 3	37 (70)	60 (14)
Metastatic site		
Liver	36 (50)	365 (85)
Lung	20 (28)	153 (35)
Peritoneum	6 (8)	19 (4)
Skeleton	2 (3)	
Pleura	2 (3)	
Adrenal gland	2 (3)	
Muscle	1 (1)	
Pericardium	1 (1)	
Kidney	1 (1)	
Intestinal mesenterium	1 (1)	
Previous chemotherapy		
Neoadjuvant	3 (4)	–
Adjuvant	24 (32)	–
Concomitant with radiation	0 (0)	23 (5)
Previous Radiotherapy	0 (0)	19 (4)

Number (%) were not else stated. *SE* South Eastern Region of Sweden

**Fig. 2:**
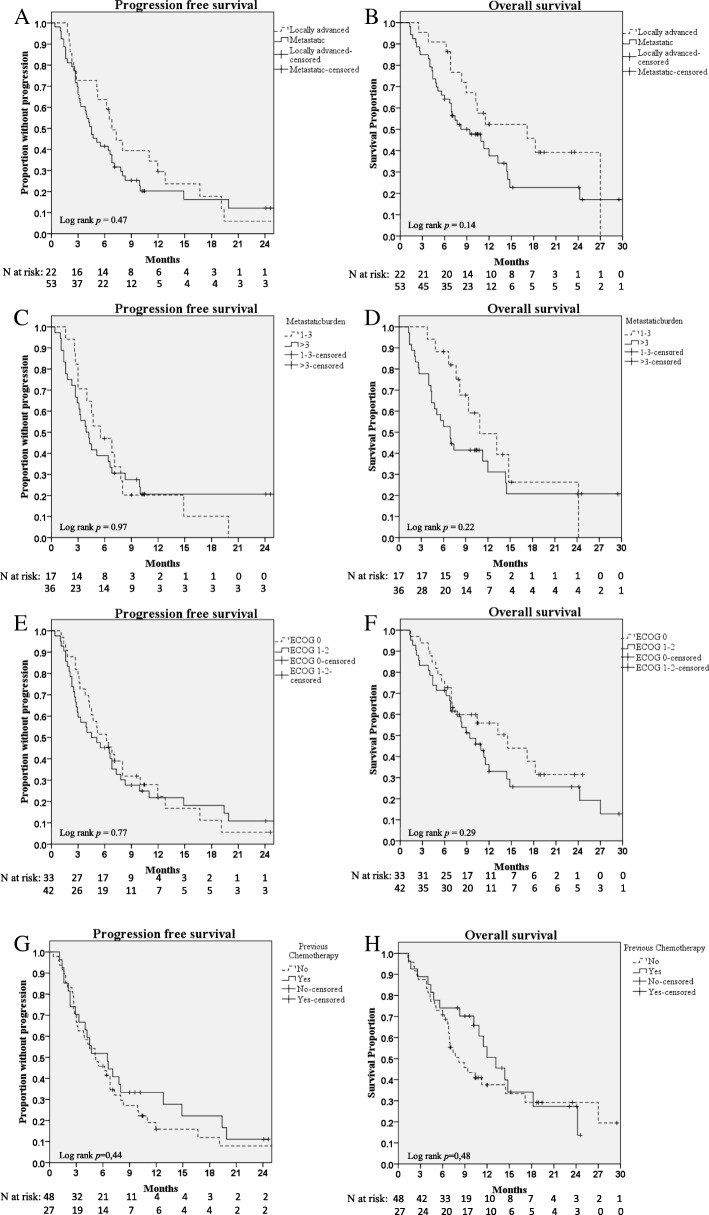
Kaplan-meier diagrams showing PFS (left column) and OS (right column) for subgroups according to stage (**a**, **b**), metastatic burden (**c**, **d**), performance status (**e**, **f**), and a previous history of non-palliative chemotherapy (**g**-**h**). *P*-values for log rank test are shown in each panel

For the entire cohort the median PFS was 5.2 months (3.4–7.0 95% CI) and the median OS was 10.9 months (7.8–14.0 95% CI, Table 2). Median follow-up was 11.2 months (median time on study among event-free/alive at end of follow-up). At time of analysis 63 patients (84%) had progressive disease and 48 patients (64%) had died. Results of the univariate analysis are shown in Table 2, Fig. 2, and Additional file 1: Figure S1, with subgroups according to locally advanced or metastasised disease, metastatic burden (more or less than three metastases), performance status 0 or 1–2, previous vs. no previous non-palliative chemotherapy, and previous vs. no previous surgery. For the mentioned subgroups there was a trend towards better prognosis in the locally advanced group with median OS 17.1 months (7.6–26.6) compared to 9.4 months (4.9–13.9) in the metastasised group although the difference was not significant (*p* = 0.14)(Fig. 2b). Similarly PFS was slightly better in the locally advanced vs. metastatic disease subgroup with a median PFS of 6.8 (5.2–8.4) and 4.5 (3.3–5.7) months, respectively (*p* = 0.47)(Fig. 2a). No significant differences were evident in subgroups with low vs. high metastatic burden, and ECOG 0 vs. 1–2 (Table 2, Fig. 2c-f). Subgroup analysis in patients who had vs. had not received previous adjuvant/neoadjuvant chemotherapy revealed slightly better OS and PFS among those who had received previous chemotherapy, although these differences were not significant (Table 2 and Fig. 2g-h). A similar subgrouping based on ‘previous surgery’ vs. ‘no previous surgery’ revealed similar results (Table 2 and Additional file 1: Figure S1). Baseline characteristics in these subgroups are found in Additional file 2: Table S1. Due to site-specific differences in blood sampling, comparative analyses of factors such as CA-19.9 and neutrophil-lymphocyte-ratio were not possible.

**Table 2:** Survival data and univariate analyses

	med PFS	HR (95% CI)	med OS	HR (95% CI)
SE-Region	5.2 (3.4–7.0)	–	10.9 (7.8–14.0)	–
MPACT (Gem/Nab-arm)	5.5 (4.5–5.9)	–	8.5 (7.9–9.5)	–
Subgroup analysis				
Tumour stage				
Locally adv.	6.8 (5.2–8.4)	0.82 (0.48–1.40) *p* = 0.47	17.1 (7.6–26.6)	0.62 (0.32–1.18) *p* = 0.14
Metastasised	4.5 (3.3–5.7)		9.4 (4.9–13.9)	
Metastatic burden				
1–3 metastases	5.5 (2.5–8.5)	0.99 (0.53–1.86) *p* = 0.97	10.9 (5.5–16.3)	0.63 (0.30–1.32) *p* = 0.22
> 3 metastases	3.9 (2.4–5.4)		6.9 (4.8–9.0)	
ECOG				
0	6.2 (3.9–8.5)	0.93 (0.56–1.54) *p* = 0.77	14.5 (7.5–21.5)	0.73 (0.40–1.31) *p* = 0.29
1–2	4.5 (1.1–7.9)		9.4 (6.3–12.5)	
Prior chemotherapy				
Yes	6.5 (2.9–10.1)	0.82 (0.48–1.38) *p* = 0.45	13.2 (9.4–17.0)	0.81 (0.44–1.46) *p* = 0.48
No	5.1 (3.0–7.2)		8.2 (5.3–11.1)	
Prior Surgery				
Yes	5.5 (2.1–8.9)	0.89 (0.53–1.50) *p* = 0.67	12.0 (9.0–15.0)	0.87 (0.48–1.58) *p* = 0.64
No	5.1 (2.8–7.4)		8.9 (5.3–12.5)	

Survival data in months (95% CI). Upper part shows data for the entire cohort compared to the MAPCT-trial. Lower part displays univariate analyses in the respective subgroups. *HR* hazard ratio calculated using Cox regression analysis

Table 3 shows frequencies of bone marrow toxicity observed according to the National Cancer Institute Common Terminology Criteria for Adverse Events (CTCAE), version 4 (CTCAE v3 used in MPACT generated the same result). The frequency of grade 3–4 bone marrow toxicity in this material was 4% for anaemia, 20% for leukopenia, 23% for neutropenia, and 5% for thrombocytopenia. Again, data from the MPACT [13] phase III trial are shown for comparison. Febrile neutropenia was recorded in 3 patients (4%) in this study compared to 14 patients (3%) in MPACT. As seen in Table 4 dose reduction was necessary in 80% of cases and there was no significant difference between gemcitabine and nab-paclitaxel. The most frequent reasons for dose reduction, as given in medical records, were neutropenia, comorbidity, infection, neuropathy, and thrombocytopenia. Average dose intensity during the whole treatment (as percentage of full dose calculated from individual body surface area) was 69% (18–100) for nab-paclitaxel and 78% (33–100) for gemcitabine. The median number of treatment cycles was 4 [1–20].

**Table 3:** Hematologic toxicity

Toxicity grade	0	1	2	3	4	SE Region 3–4	MPACT 3--4
Anemia	4 (5)	32 (43)	36 (48)	3 (4)	0 (0)	3 (4)	53 (13)
Leukopenia	31 (41)	12 (16)	17 (23)	14 (19)	1 (1)	15 (20)	124 (31)
Neutropenia	45 (60)	3 (4)	10 (13)	11 (15)	6 (8)	17 (23)	153 (38)
Thrombocytopenia	18 (24)	42 (56)	11 (15)	4 (5)	0 (0)	4 (5)	52 (13)

Occurrence of bone marrow toxicity according to CTCAE version 4, n (%)

**Table 4:** Treatment data

	SE Region
Occurrence of dose reduction	
Gemcitabine	60 (80)
Nab-Paclitaxel	60 (80)
Reason given for dose reduction	
Neutropenia	23 (26)
Comorbidity	13 (15)
Infection	11 (12)
Neuropathy	11 (12)
Thrombocytopenia	8 (9)
Liver toxicity	6 (7)
Anaemia	5 (6)

Dose reduction and reasons for dose reduction, n(%) where not else stated. *SE* South Eastern Region of Sweden

Second line therapy was given to 51% of patients as listed in Table 5. Most common second line regimen was FLOX (5-FU bolus/Leukovorin/Oxaliplatin). In addition to standard chemotherapy one patient received Hyperthermic Intra Peritoneal Chemotherapy (HIPEC), one patient received Radiofrequency Ablation of liver metastases (RFA), and two patients received Irreversible Electroporation (IRE) treatment. Although the low numbers did not allow for specific subgroup analyses in these patients, median OS in the total cohort was similar regardless of the inclusion or exclusion of these four patients (Additional file 3: Figure S2 and Additional file 4: Table S2).

**Table 5:** Treatment beyond progression

	2nd line	3rd line	4th line
Cap	3 (5)	1 (2)	0 (0)
FLOX	16 (27)	0 (0)	0 (0)
FLIRI	1 (2)	1 (2)	0 (0)
FLV	3 (5)	0 (0)	0 (0)
FOLFIRINOX	4 (7)	0 (0)	0 (0)
Gem	0 (0)	0 (0)	1 (2)
GemCap	2 (3)	4 (7)	0 (0)
GemOX	1 (2)	0 (0)	0 (0)
No treatment	29 (49)	53 (89)	58 (98)

Therapy lines following first line treatment with Gem/NabP, n (%). Abbreviations: *Cap* Capecitabine, *FLOX* 5-fluorouracil (bolus) + folinic acid + oxaliplatin, *FLIRI* 5-fluorouracil (bolus) + folinic acid + irinotecan, *FLV* 5-fluorouracil (bolus) + folinic acid, *FOLFIRINOX* 5-fluorouracil (bolus and continuous infusion) + folinic acid + irinotecan + oxaliplatin, *Gem* Gemcitabine, *GemCap* Gemcitabine + Capecitabine, *GemOX* Gemcitabine + Oxaliplatin

## Discussion

This retrospective study on Swedish patients with advanced pancreatic cancer provides real world evidence of treatment benefit with Gem/NabP with outcome results similar to those reported in the MPACT phase III trial [13]. To our knowledge this study offers the most mature real world data on this treatment indication and regimen so far. Due to key differences in study designs and inclusion/exclusion criteria, a direct statistical comparison between the MPACT and the present real world population was not feasible, but notably median PFS and OS were comparable in the respective populations, with completely overlapping confidence intervals, indicating that Gem/NabP is at least as effective and generally well tolerated in the real world compared with the randomised controlled trial setting. When relating the results to MPACT there are some differences in patient characteristics that need to be taken into account. The patients included in MPACT were younger, with a substantial portion of patients being less than 65 years. They were also chemo-naïve as opposed to the 35% of our patients that had previous non-palliative therapy with predominantly adjuvant gemcitabine. The latter subgroup appeared to have slightly better survival in the present real world population although no significant differences in PFS or OS between those who received, versus those who did not receive, previous non-palliative chemotherapy were evident (Table 2 and Fig. 2g-h). Notably, median PFS and OS were almost identical in the ‘no previous chemotherapy’ group of our study and the MPACT population. A similar kind of comparison between those who had, versus those who had not, undergone previous pancreatic surgery yielded similar results (Table 2 and Additional file 1: Figure S1).

Notably, in this study cohort 29% were diagnosed with locally advanced disease without signs of distant metastases whereas the MPACT trial only included patients with upfront metastatic disease. The subgroup analysis on disease stage shows that the metastasised patients in our cohort actually has a slightly longer estimated median OS than the median OS observed in the MPACT trial (9.4 vs 8.9 months, Table 2, Fig. 2b). On the other hand, the fraction of patients with more than three metastases was very low compared to our cohort (14 vs 70%). The subgroup analysis on metastatic burden indicates a better prognosis for the patients with less than three metastases although this difference was not statistically significant (median OS 10.9 vs 6.9 months, *p* = 0.22)(Table 2, Fig. 2d).

There are two larger previous retrospective studies on this topic. Braiteh et al utilized time to discontinuation (TTD) as a surrogate for PFS and database persistence as a surrogate for OS, in a Celgene corporation funded study including 122 patients [14], and reported numbers of 3.4 and 8.6 months for these respective parameters. Kim et al revealed a TTD of 4.3 months in a study of 182 patients focusing on healthcare economics, reporting no OS data [15]. Lo Re et al reported survival data similar to ours in a smaller previous study of 37 patients [16], while Montes et al found slightly longer PFS and OS of 9 and 15 months respectively in a retrospective study of 39 patients [17]. In another recent retrospective study by Wang et al [18], where both metastatic and locally advanced patients were included, similar survival measures were observed with 10.5 months overall survival in the total cohort and 10.0 months in a metastatic subgroup. However, follow up time was not sufficient to record median OS in the locally advanced subgroup.

This study provides additional value since it was performed on a reasonably large population, with robust recognition of clinico-pathological and treatment parameters and sufficient follow up times to analyse measured (and not estimated or surrogate) primary and secondary endpoints. Mature data on the locally advanced subgroup, that was not included in the MPACT trial, is further provided and encourages further usage of the combination regimen in this subgroup. The Swedish general and public funded health care system means that all individuals regardless of socio-economic status, insurance status, or comorbidities are offered similar treatments and follow-up programs, and the present study therefore adds key information about the outcome and tolerability of Gem/NabP in a truly ‘real world’ setting.

The patients in the present study experienced less hematologic grade 3–4 toxicity than in the MPACT study, and in the lower interval of that reported in previous studies [14, 16, 17]. As chemotherapy dosage data have not been published in these studies we cannot distinguish if this difference is attributable to differences in dose intensity. Our data was based on routine blood sampling prior to each dose of chemotherapy. Neutrophil count was not tested as routine at some centres which means that neutropenia is presumably underestimated in this study. The vast majority of patients (80%) needed chemotherapy dose reduction, underlining the need for biomarkers or clinical features predicting toxicity. Notably a substantial part (51%) of the patients in this study were subjected to second line therapy, mostly 5-FU with folinic acid alone or in combination with oxaliplatin. The corresponding figure in MPACT trial was 38%. The proportion of patients receiving 5-FU/oxaliplatin combination was quite high in our population, which probably reflects a standard of care that was inspired by the CONKO-003 [19] trial but not taking into account the more recent PANCREOX trial [20] that displayed no benefit of oxaliplatin combination compared to 5-FU/Leucovorin alone. In previous real world studies second line treatment was only described by Braiteh et al., who reported a frequency of 20% [14].

## Conclusion

This study confirms the effectiveness and safety of Gem/NabP in both locally advanced and metastasised PDA in a real world setting. Less treatment related haematological toxicity than expected was observed. The vast majority of patients underwent dose-adjustments. Biomarkers predicting effects and toxicity of Gem/NabP are still lacking, and prospective trials investigating and validating serum- or tissue markers are needed in order to establish tools for personalised treatment decisions.

## Additional files

Additional file 1: **Figure S1.** Kaplan-Meier diagrams showing PFS (A) and OS (B) for subgroups according to the occurrence of prior curative intent pancreatic resection. (PDF 124 kb)

Additional file 2: **Table S1.** Baseline patient characteristics for subgroups divided according to previous chemotherapy treatment and previous curative intent surgery, respectively. (DOCX 19 kb)

Additional file 3: **Figure S2.** Kaplan-Meier diagram showing OS for the entire cohort with or without the inclusion of four patients who received unconventional/experimental treatment (HIPEC [*n* = 1], RFA [*n* = 1], and IRE [*n* = 2]) in addition to Gem/NabP. (PDF 100 kb)

Additional file 4: **Table S2.** Survival data in months (95% CI) for the entire cohort compared to patients that received standard treatment only. HR = hazard ratio calculated using Cox regression analysis. Experimental treatments were RFA (*n* = 1), HIPEC (*n* = 1), and IRE (*n* = 2). (DOCX 14 kb)

## Abbreviations

aPDA: Advanced pancreatic ductal adenocarcinoma
CTCAE: National Cancer Institute Common Terminology Criteria for Adverse Events
Gem/NabP: Gemcitabine and nab-paclitaxel
HIPEC: Hyperthermic Intra Peritoneal Chemotherapy
IRE: Irreversible Electroporation
OS: Median overall survival
PFS: Median progression free survival
RFA: Radiofrequency Ablation
TTD: Time to discontinuation
