# Supplementary figures and images for: Real world evidence on gemcitabine and nab-paclitaxel combination chemotherapy in advanced pancreatic cancer
Source: BMC Cancer. 2019 Jan 8;19:40. doi: 10.1186/s12885-018-5244-2 (PMC6325739; doi:10.1186/s12885-018-5244-2)

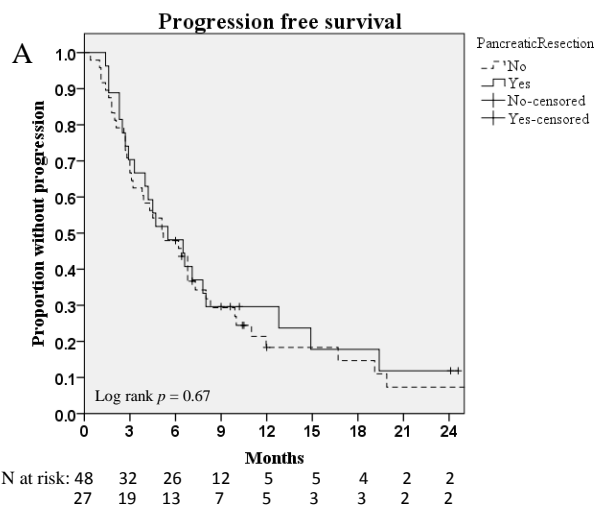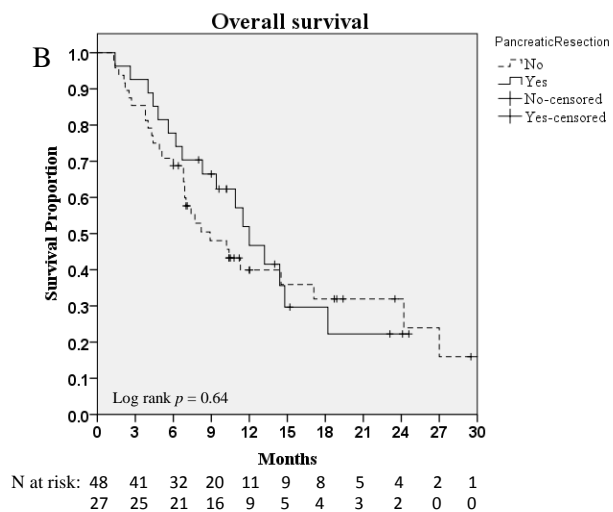

Supplement: Supplementary file 1 — Figure S1. Kaplan-Meier diagrams showing PFS (A) and OS (B) for subgroups according to the occurrence of prior curative intent pancreatic resection. (PDF 124 kb) [file 12885_2018_5244_MOESM1_ESM.pdf]

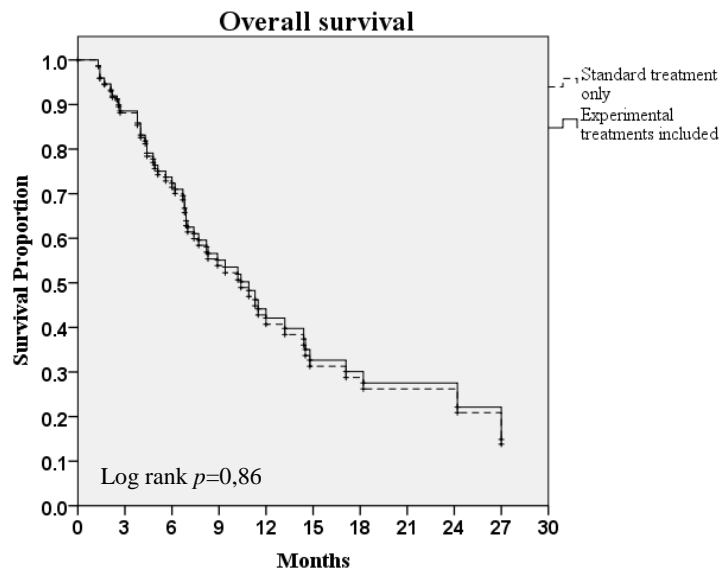

Supplement: Supplementary file 3 — Figure S2. Kaplan-Meier diagram showing OS for the entire cohort with or without the inclusion of four patients who received unconventional/experimental treatment (HIPEC [n = 1], RFA [n = 1], and IRE [n = 2]) in addition to Gem/NabP. (PDF 100 kb) [file 12885_2018_5244_MOESM3_ESM.pdf]
