# Supplementary material for: Real world evidence on gemcitabine and nab-paclitaxel combination chemotherapy in advanced pancreatic cancer
Source: BMC Cancer. 2019 Jan 8;19:40. doi: 10.1186/s12885-018-5244-2 (PMC6325739; doi:10.1186/s12885-018-5244-2)
Supplement: Supplementary file 2 — Table S1. Baseline patient characteristics for subgroups divided according to previous chemotherapy treatment and previous curative intent surgery, respectively. (DOCX 19 kb) [file 12885_2018_5244_MOESM2_ESM.docx]

**Table S1**.

|  | **SE adj/neoadj chemotherapy** | **SE no prior chemotherapy** | **SE Region surgery** | **SE Region**  **no surgery** |
| --- | --- | --- | --- | --- |
| Total number | 27 (100) | 48 (100) | 27 (100) | 48 (100) |
| Gender |  |  |  |  |
| Female | 11 (41) | 23 (48) | 12 (44) | 22 (46) |
| Male | 16 (59) | 25 (52) | 15 (56) | 26 (54) |
| Age median(range) | 68 (51-80) | 65 (48-76) | 68 (48-80) | 65 (50-76) |
| Distribution |  |  |  |  |
| <65 median | 8 (30) | 21 (44) | 8 (30) | 21 (44) |
| ≥65 median | 19 (70) | 27 (56) | 19 (70) | 27 (56) |
| Length cm median(range) | 173 (154-191) | 169 (150-192) | 172 (154-191) | 171 (150-192) |
| Weight kg median(range) | 69 (41-97) | 74 (47-121) | 69 (56-97) | 75 (47-121) |
| Body surface kg/m^2^  median(range) | 1.86 (1.4-2.15) | 1.88 (1.4-2.37) | 1.81 (1.4-2.15) | 1.88 (1.4-2.37) |
| ECOG Performance status |  |  |  |  |
| 0 | 9 (33) | 24 (50) | 9 (33) | 24 (50) |
| 1 | 17 (63) | 19 (40) | 17 (63) | 19 (40) |
| 2 | 1 (4) | 5 (10) | 1 (4) | 5 (10) |
| CA19-9 U/ml median(range) | 191 (1-140000) | 739 (1-91928) | 183 (1-9200) | 756 (1-140000) |
| Tumour stage |  |  |  |  |
| Locally advanced | 5 (19) | 17 (35) | 5 (19) | 17 (35) |
| Metastasised | 22 (81) | 31 (65) | 22 (81) | 31 (65) |
| Number of metastases |  |  |  |  |
| 1 | 6 (27) | 2 (6) | 6 (27) | 2 (6) |
| 2 | 1 (5) | 5 (16) | 2 (9) | 4 (13) |
| 3 | 2 (9) | 0 (0) | 2(9) | 0 (0) |
| >3 | 13 (59) | 24 (77) | 12 (55) | 25 (81) |
| Metastatic site |  |  |  |  |
| Liver | 12 (41) | 24 (56) | 12 (44) | 24 (55) |
| Lung | 10 (34) | 10 (23) | 9 (33) | 11 (25) |
| Peritoneum | 2 (7) | 4 (9) | 2 (7) | 4 (10) |
| Skeleton | 1 (3) | 1 (2) | 1 (4) | 1 (2) |
| Pleura | 1 (3) | 1 (2) | 1 (4) | 1 (2) |
| Adrenal gland | 1 (3) | 1 (2) | 1 (4) | 1 (2) |
| Muscle | 1 (3) | 0 | 1 (4) | 0 (0) |
| Pericardium | 1 (3) | 0 | 1 (4) | 0 (0) |
| Kidney | 0 | 1 (2) | 0 (0) | 1 (2) |
| Intestinal mesenterium | 0 | 1 (2) | 0 (0) | 1 (2) |
| Previous chemotherapy |  |  |  |  |
| Neoadjuvant | 3 (11) | 0 (0) | 0 (0) | 3 (6) |
| Adjuvant | 24 (89) | 0 (0) | 24 (89) | 0 (0) |
| Concomitant with radiation | 0 (0) | 0 (0) | 0 (0) | 0 (0) |
| Previous Radiotherapy | 0 (0) | 0 (0) | 0 (0) | 0 (0) |

**Table S1**. Baseline patient characteristics for subgroups divided according to previous chemotherapy treatment and previous curative intent surgery, respectively.
