# Supplementary material for: Real world evidence on gemcitabine and nab-paclitaxel combination chemotherapy in advanced pancreatic cancer
Source: BMC Cancer. 2019 Jan 8;19:40. doi: 10.1186/s12885-018-5244-2 (PMC6325739; doi:10.1186/s12885-018-5244-2)
Supplement: Supplementary file 4 — Table S2. Survival data in months (95% CI) for the entire cohort compared to patients that received standard treatment only. HR = hazard ratio calculated using Cox regression analysis. Experimental treatments were RFA (n = 1), HIPEC (n = 1), and IRE (n = 2). (DOCX 14 kb) [file 12885_2018_5244_MOESM4_ESM.docx]

**Table S2.**

|  | **med PFS** | **HR (95% CI)** | **med OS** | **HR (95% CI)** |
| --- | --- | --- | --- | --- |
| **Subgroup analysis** |  |  |  |  |
| Experimental treatments |  |  |  |  |
| Excluded | 5.5 (3.5-7.5) | 1.02 (0.72-1.46) *p*=0.90 | 10.4 (7.1-13.8) | 0.96 (0.64-1.64) *p*=0.86 |
| Included | 5.2 (3.4-7.0) |  | 10.9 (7.8-14.0) |  |

**Table S2.** Survival data in months (95% CI) for the entire cohort compared to patients that received standard treatment only. HR = hazard ratio calculated using Cox regression analysis. Experimental treatments were RFA (n=1), HIPEC (n=1), and IRE (n=2).
